# How to Reduce the Risk of Mechanical Failures in Adult Deformity Surgery: Comparing GAP Score and Roussouly Type Restoration

**DOI:** 10.1177/21925682251328285

**Published:** 2025-03-20

**Authors:** Domenico Compagnone, Riccardo Cecchinato, Andrea Pezzi, Francesco Langella, Marco Damilano, Daniele Vanni, Andrea Redaelli, Claudio Lamartina, Pedro Berjano

**Affiliations:** 1 Department of GSpine 4, IRCCS Ospedale Galeazzi-Sant’Ambrogio, Milan, Italy; 2 Department of Biomedical Sciences for Health, 9304 University of Milan, Milan, Italy

**Keywords:** adult spinal deformity, surgical complications, mechanical failures, GAP Score, Roussouly classification, 3 retrospective cohort study

## Abstract

**Study Design:**

Retrospective Cohort Study.

**Objectives:**

To assess long-term alignment descriptors correlating with mechanical complications.

**Methods:**

The study included adult spinal deformity cases older than 18, with a minimum of four instrumented levels and a 5-year follow-up. Exclusions: previous spinal fusion, neuromuscular/rheumatic diseases, active infections, tumors, or incomplete radiographic exams. Collected data: demographic, surgical, pre- and post-operative spinopelvic parameters, and post-operative complications. The GAP score, original Roussouly type restoration, Schwab’s criteria, and Odontoid to hip axis angle were evaluated using machine learning and logistic regression. Complications were evaluated with a Kaplan-Meier curve.

**Results:**

Two hundred and twelve patients fulfilled the inclusion and exclusion criteria and were enrolled in the study. The observed rate of revision surgery for mechanical complications was 40.6% (86 out of 212 patients). Higher post-operative GAP scores were associated with increased risks of revision for junctional failure (AUC = 0.72 [IC 95%] 0.62-0.80). The inability to restore the original Roussouly spinal shape was statistically associated with higher mechanical failure rates. A machine-learning approach and subsequent logistic regression found that the GAP score and original Roussouly type restoration are the most important predictors for mechanical failure, and GAP score lordosis distribution index and relative pelvic version are the most important factors to predict the risk of mechanical failure.

**Conclusions:**

In our series, a proper post-operative GAP Score and the restoration of the original Roussouly type significantly minimize mechanical complication rates. We observed that junctional failure tends to occur earlier among complications, while implant failure occurs later in the follow-up.

## Introduction

Adult spinal deformity (ASD) is a disruption of the spine’s normal alignment in the coronal and sagittal planes that occurs in the adult population, with a prevalence ranging from 32% to 68% among individuals over 65 years old.^1^ In subjects unresponsive to conservative treatment, it has been demonstrated that the surgical approach significantly increases the health-related quality of life.^2^ Although many authors recognize proper post-operative alignment as the cornerstone of the surgery, given its impact on clinical outcomes^3^ and the risk of mechanical complications (MC),^4^ a definition of good sagittal balance is still debated. According to the Scoliosis Research Society (SRS)-Schwab classification,^5^ the surgical targets needed to achieve a satisfactory alignment are:

• Pelvic Incidence (PI) minus Lumbar Lordosis (LL) ≤ ±9°
• Pelvic Tilt (PT) < 20°
• Sagittal Vertical Axis (SVA) < 4 cm

Although these parameters significantly correlate with disability and quality of life indicators, there is poor evidence regarding their ability to forecast MC.^6^ Indeed, even patients who postoperatively respect these targets showed a high rate of mechanical failure (31.7%), confirming the need for a deeper analysis.^7^

The concept of lordosis distribution, not considered by the SRS classification, is fundamental in the Roussouly classification,^8^ where the different shapes of the spine have different lordosis distribution and length. Several authors, including Roussouly himself, proposed that ignoring the restoration of the original spinal type is associated with an increased risk of mechanical complications.^8^ The main criticism levelled at these studies is the difficulty in evaluating the original Roussouly type in a deformity scenario and the absence of clarity in the adopted criteria.

The Global Alignment and Proportion (GAP) Score, published in 2017 by Yilgor et al,^9^ tries to unify the evaluation of pelvic orientation, lordosis distribution and global spinal alignment, with the claim to represent a comprehensive alignment parameter able to predict mechanical complications after ASD surgery.

Our retrospective study aims to verify the most reliable parameters for predicting the rate of MC and clarify the alignment targets for preventing its occurrence.

## Materials and Methods

### Study Design and Patients

This study followed the STROBE cohort reporting guidelines^10^; it was a retrospective cohort analysis of cases of ASD correction in a high-volume division with 5 years minimum follow-up. This study was conducted from an institutional spine surgery registry — SpineReg. The study was approved by the ethics committee of the San Raffaele hospital - Milan IRCCS (substantial amendment no. 3 of 05/09/2019 given the previous approval to the SPINEREG register with the number of the register of opinions of the ethics committee 93/INT/2015), and every patient signed a written consent before their data joined the registry.

The inclusion criteria were:

• Patients older than 18;
• Posterior instrumented fusion of at least 4 vertebrae;
• A minimum follow-up of 5 years with complete radiological data.
• ASD cases were defined as patients with at least one of the following radiological parameters before surgery:
  ▪ Scoliosis Cobb angle >20°;
  ▪ Sagittal Vertical Axis (SVA) > 5 cm;
  ▪ Thoracic Kyphosis (TK) > 60°;
  ▪ Pelvic Tilt (PT) ≥ 20°.

Patients were excluded from analysis if they received other previous spine fusion procedures, were diagnosed with post-traumatic deformities, neuromuscular diseases, rheumatic diseases, active infections, or tumors, or if clinical and radiological data from pre-operative, early post-operative (between 0 and 12 weeks), and at least 5 years after the surgery were unavailable.

### Data Collection

Eligible patients were identified from the Institute’s clinical database, containing surgical reports, from 2008 to 2016. All patients were enrolled in accordance with inclusion and exclusion criteria.

All the surgical procedures performed in each patient were reviewed.

For all the patients, radiographic and clinical variables were collected pre-operatively and immediately post-operatively (within the first 12 weeks). All the x-rays were evaluated before the revision surgery, when needed. At the last-FU (minimum 5 years), the patients were evaluated with a full-spine x-ray or on an outpatient basis to provide information on any complication or subsequent spinal surgery performed in our or other hospitals.

**Figure 1.**
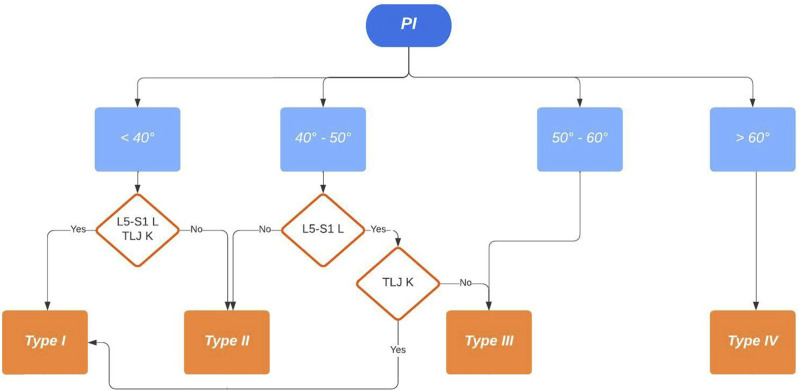
Preoperative Roussouly type classification in deformity. PI – pelvic incidence; L5-S1 L – lordotic L5-S1; TLJ K – kyphotic thoraco-lumbar junction.

**Figure 2.**
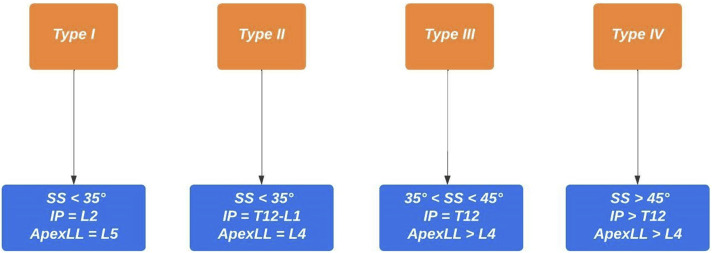
Postoperative restoration of Roussouly type. SS – sacral slope; IP – inflection point; ApexLL – apex of lumbar lordosis.

The radiographic measurements were performed using a properly designed and validated software (Surgimap Spine Software, Nemaris Inc., NY, USA).^11^ Patients with radiographic examinations that did not meet the protocol criteria were excluded from the study.^12^ All measurements were performed in the same hospital by two authors, experienced spine surgeons (D.C. and A.P), with a good inter- and intraobserver agreement. Preoperative and postoperative Roussouly type classification was performed by two of the authors according to Figures 1 and 2 (D.C and A.P) and all disagreements were solved by a meeting held in consultation with a third senior author (R.C).

All data regarding the surgical revisions due to mechanical failure were recorded.

### Study Variables

#### Cohort Descriptive Variable

All patients’ demographic variables, including age at the time of surgery and sex, were collected before the surgical procedure. Surgical techniques and instrumented levels data were also collected.

For radiographic variables, we measured PI, PT, sacral slope (SS), maximum LL, lordosis between L4 and S1 (LLL), thoracic kyphosis (TK), the SVA, Global Tilt (GT), Odontoid and the center of the hip axis angle (ODHA), Cobb degrees of the scoliotic curve preoperatively and in the early post-operative (between 0 and 12 weeks). We evaluated the inflection point (IP) and the apex of lordosis (ApexLL) preoperatively and early postoperative.

#### Predictor Variables

We considered the GAP score, calculated as described by Yilgor et al,^9^ the restoration of the original Roussouly type, PI-LL, SVA, ODHA, and the adherence to Schwab’s criteria as independent variables.

The population was stratified according to the following postoperative analysis:

• GAP score: We created a Receiver Operator Characteristic (ROC) curves that showed a GAP score threshold value that optimized the relationship between sensitivity and specificity (higher Youden’s index = [sensitivity - (1-specificity)]) = 4 (Youden’s Index = 1.37); we considered: patients with GAP score ≤4 as indicative of proportioned balance (GAP-P); patients with GAP score >4 as disproportionate spinopelvic balance (GAP-D).
• Roussouly type restoration: the preoperative type of Roussouly was identified according to Figure 1. Roussouly type was considered as restored when, postoperatively, at least two out of the three parameters shown in Figure 2 were within the expected range for the corresponding Roussouly type;
• Schwab’s Criteria: The adherence to Schwab’s criteria was assessed when postoperative PI-LL ≤ ± 10°, PT < 20° and SVA <4 cm;
• ODHA: Patients with postoperative ODHA between −5 and +2 was considered as aligned;
• SVA: Patients with postoperative SVA <4 cm were considered as aligned.

GAP score, Roussouly type restoration, Schwab’s Criteria, ODHA and SVA have been evaluated in a binary fashion to assess the target achieved (1) or not achieved (0), to provide data for the Machine Learning and Logistic Regression.

We independently analyzed the subparts of each variable: for the GAP score, the age of the patient (AGE), the postoperative relative pelvic version (RPV), relative lumbar lordosis (RLL), lordosis distribution index (LDI), and relative spinopelvic alignment (RSA) according to Yilgor
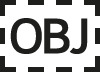
; for the restoration of the original Roussouly type, the ApexLL, the SS, and the IP.

#### Outcomes Dependent Variables

We estimated the rate of clinically relevant MC, defined as any mechanical complication that needs surgery.

We categorized clinically-relevant MC into complications related to the implant (implant failures IF), such as breakage of the rods, loosening, pull-out or rupture of screws or interbody cages, fixation elements loss of anchorage, non-union), and junctional failures (JF), divided in proximal junctional failure (PJF), and distal junctional failure (DJF). PJF is defined as the occurrence of the upper instrumented vertebra (UIV) or UIV +1 fracture, pull-out of UIV instrumentation, presence of sagittal subluxation or symptomatic proximal junctional kyphosis; DJF is described by the lower instrumented vertebra (LIV) or LIV +1 fracture, pull-out of the LIV instrumentation, symptomatic distal junctional kyphosis that needs revision surgery.

### Statistical Analysis

Data were analyzed using SPSS version 21.0 (SPSS Inc., Chicago, IL, USA). Demographics and radiographic parameters were presented using descriptive statistics. Categorical data were presented with absolute frequencies and percentages, and continuous variables were expressed as mean ± standard deviation.

In the current investigation, supervised Machine Learning (ML) technology was applied to examine the relationship between all the analyzed variables. ML, particularly supervised binary classification, involves pattern recognition in available data to make informed predictions about future events, where the desired output labels are known. Specifically, Gradient Boosting as best and performant model was used to infer predicted features related to the MC.

Advanced machine learning (ML) techniques allowed for a comprehensive analysis of postoperative outcomes. The primary objective was identifying the parameters and variables that significantly influence the final outcome. The study applies a binary classification method, with ‘Complication’ as the target variable. Powerful ML approaches were implemented for predictive analysis. To fulfill the analytic objective, three distinct models were applied: Decision Trees, Random Forests and Gradient Boosting. For each model the Average Squared Error (ASE) was assessed:

- Decision Trees: ASE 0.158
- Random Forests: ASE 0.156
- Gradient Boosting: ASE 0.073

A logistic regression analysis was also performed to investigate the factors that reached statistical significance and impacted the MC rate.

ROC curves were used to evaluate the association between the GAP score and the need for revision surgery to treat an MC. An area under the curve (AUC) of 0.5 to 0.7 was classified as no or low associative power, 0.7 to 0.9 as moderate, and >0.9 as high. Besides, the Kaplan-Meier estimation plot assessed the post-operative GAP score’s influence on the “survival time” without mechanical failure. The log-rank test was calculated to verify any significant association.

The Kaplan-Meier estimation plot was assessed to evaluate the statistical association between surgical revision for mechanical failure and the restoration of the original Roussoul, and the log-rank test was calculated to verify any significant association.

A supervised ML technology was again applied to examine the relationship between all the subparts of the GAP score and Roussouly restoration and the occurrence of MC.

Due to the highlighted importance of postoperative GAP score and restoration of the original Roussouly type, we analysed each sub-category deeply to design a Predictive Model. In this context, the objective was to assess and estimate the variables that primarily influence the outcomes ‘complications’ (0 = no complications, 1 = complications).

The analysis was performed on 8 predictor categories, to which a risk level was assigned, with two possible scenarios:

1. [0 = no risk; 1 = high risk] for the AGE, restoration of the apex of lumbar lordosis (R_LLApex), restoration of the SS (R_SS) and the restoration of inflection point (R_IP).
2. [0 = no risk; 1 and 2 = intermediate risk; 3 = high risk] for RPV, RLL, LDI and RSA

These categorical predictors were used to assess and predict the probabilities of complications with the risk labels indicating the degree of danger associated with each predictor.

## Results

### Population

**Figure 3.**
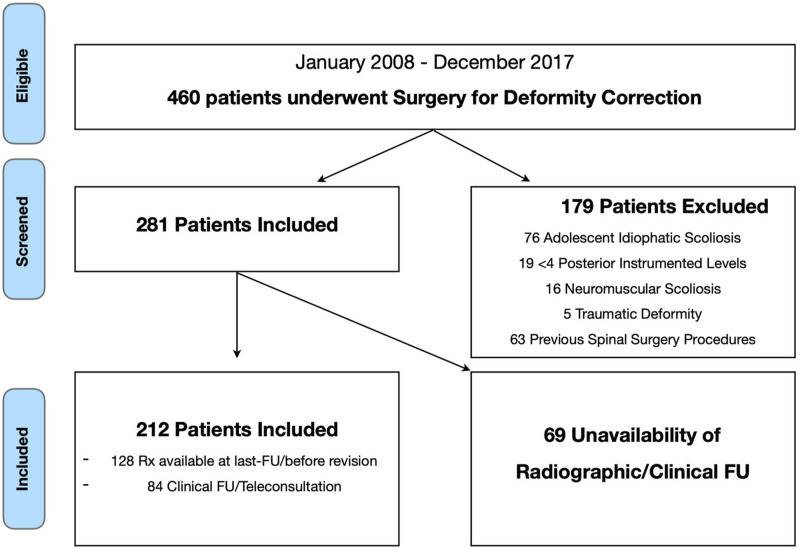
Patient selection flowchart for deformity correction surgeries.

Four hundred sixty (460) patients were treated for ASD between 2008 and 2017. Two hundred twelve (212) patients fulfilled the inclusion criteria and exclusion criteria and were definitively enrolled in the study (Figure 3). The pre-operative characteristics of the patients are presented in Table 1. No significant differences in age and gender were found between included and excluded patients (mean age: 64 ± 15 vs 61 ± 12 years; females: 85% vs 81%).

**Table 1.** Pre-Operative Characteristics of the Patients.

Variable	Mean or %	SD
Age (years)	64	15.4
Male	31	
Female	181	
FU (months)	109	28.8
N° levels fused	11	2.8
Pelvic incidence (PI)	52	12.0
Pelvic tilt (PT)	25	11.6
Sacral slope (SS)	27	13.1
Maximum lumbar lordosis (LL)	38	24.4
L4-S1 lumbar lordosis (LLL)	30	15.5
Thoracic kyphosis (TK)	38	19.7
Sagittal vertical axis (SVA)	62	63.4
Global tilt (GT)	32	18.9
Odontoid and center of hip axis angle (OD-HA)	4	3.9

### Mechanical Complications - Outcomes Dependent Variables

**Figure 4.**
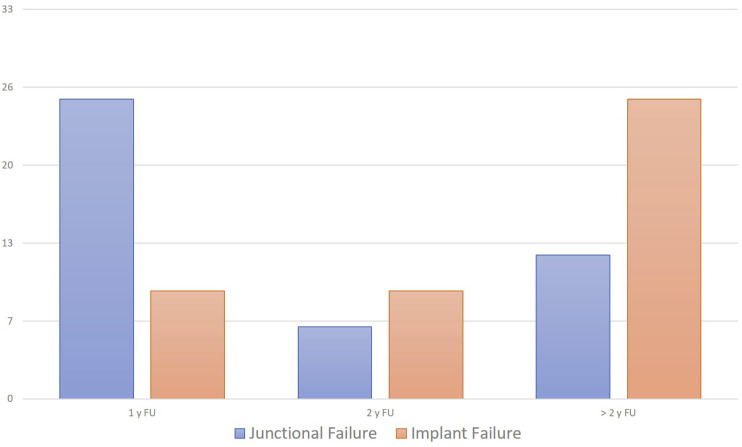
Incidence of junctional and implant failures at different follow-up intervals.

In our cohort, the revision surgery for clinically-relevant mechanical complication rate was 40.6% (86 out of 212 patients), equally shared between JF (20.3%, 43/212 patients) and IF (20.3%, 43/212 patients). Considering JF, 25 out of 43 failures (58.1%) occurred in the first year after surgery and 31 out of 43 (72.1%) within the first two years, while for IF we observed a 21% in the first year (9 out of 43) and 42% (18 out of 43) in the first two years (Figure 4).

### Multivariate Analysis - Machine Learning Approach and Logistic Regression (Step I)

**Figure 5.**
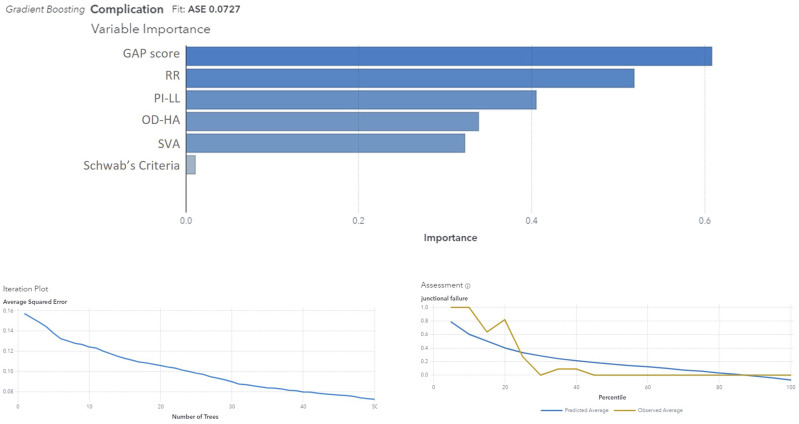
Gradient boosting analysis showing variable importance for predicting complications, with GAP score being the most significant. The iteration plot shows the average squared error decreasing with the number of trees. The assessment plot compares predicted and observed averages for junctional failure. RR - Roussouly type restoration; PI – pelvic incidence; LL – lumbar lordosis; OD-HA odontoid and center of hip axis angle; SVA - sagittal vertical axis.

The most reliable model, among all, was the Gradient Boosting model; it exhibited an exceptionally low ASE of 0.07, which underscores high performance, precise and accurate prediction power (the observed data closely aligns with the model’s predictions) and ability to provide reliable forecasts. The model’s accuracy is approximately 98%. These findings reinforce the reliability and accuracy of the gradient boosting model in predicting ‘Complication’. 70% of the cohort was needed for the Training of the model, 15% for the Validation and 15% for the Test. The importance of the variables in predicting the outcome ‘complication’ is shown in Figure 5. Table 2 presents the logistic regression analysis results, highlighting that both the GAP score and the restoration of the original Roussouly type were statistically significant predictors of mechanical complications, with the GAP score demonstrating a higher predictive value.

**Table 2.** Logistic Regression Analysis of GAP Score and Original Roussouly Type Restoration.

Variable	Coefficient (B)	SE	Odds Ratio (OR)	95% CI for OR	*P*-Value
GAPscore	0.13	0.043	1.14	1.05 – 1.24	**0.002**
RR	0.69	0.30	0.49	0.27 – 0.90	**0.042**

Bold values represents Statistically significant result.

### Univariate Analysis - GAP Score

**Figure 6.**
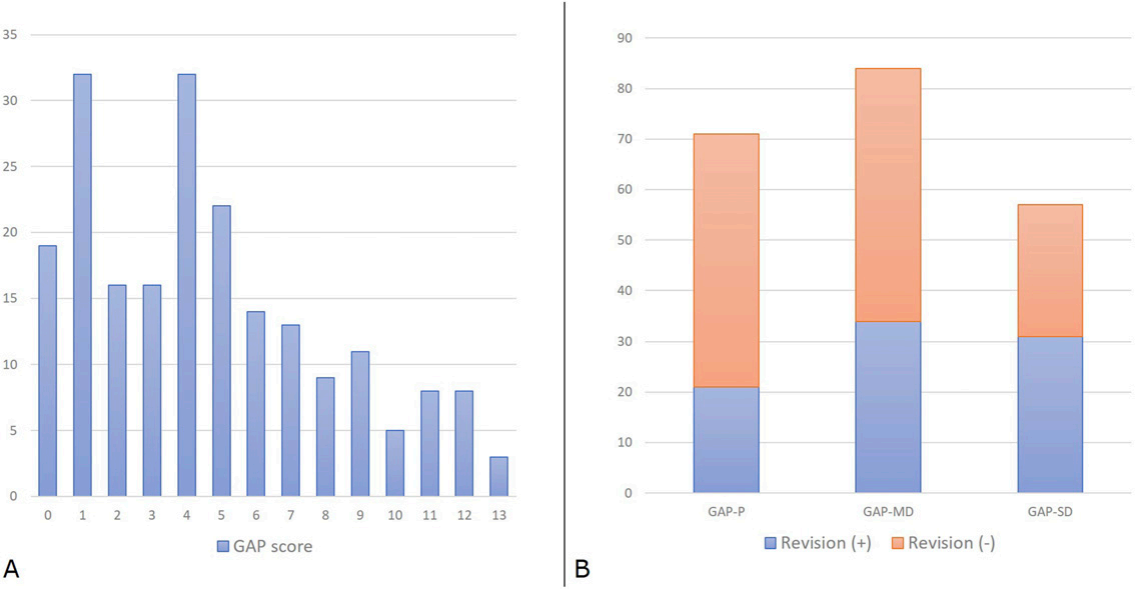
Distribution of patients (y-axis: number of patients) across GAP scores (A) and categories with revision rates for mechanical complications (B).

Figure 6(A) shows the distribution of patients across different GAP categories. Patients were stratified according to GAP score values: from 0 to 2 as indicative of proportioned balance (GAP-P); from 3 to 6 as moderately disproportioned balance (GAP-MD); ≥7 as severely disproportionate spinopelvic balance (GAP-SD). Revisions for mechanical complications were needed in 30% (21 out of 71) of patients with GAP-P, in 40.5% (34 out of 84) with GAP-MD and 54.4% (31 out of 57) with GAP-SD (Figure 6(B)).

**Figure 7.**
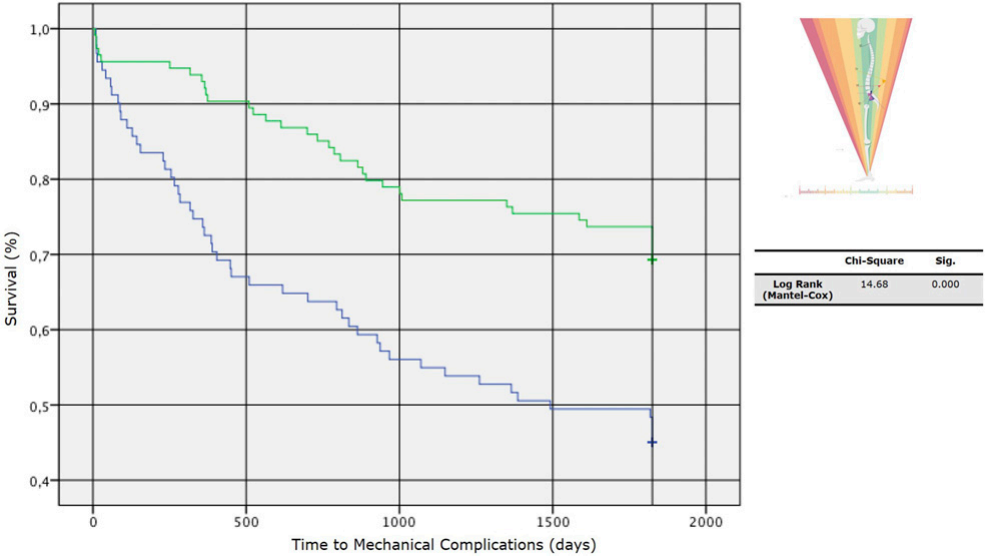
Survival rate of implants without mechanical complications (MC), comparing two subpopulations divided by a specific cut-off value.

According to the findings from the ROC curve, the GAP score threshold value that optimized the relationship between sensitivity and specificity (higher Youden’s index = [sensitivity - (1-specificity)]) was GAP score = 4 (Youden’s Index = 1.37); we divided our cohort into two subpopulations according to this cut-off value, and we analyzed the two curves describing the survival rate of implants in absence of MC (Figure 7). Log-rank tests showed statistically significant differences between survival curves with a *P* < 0.0001 - [*χ*^2^ (N = 212) = 14.68].

**Figure 8.**
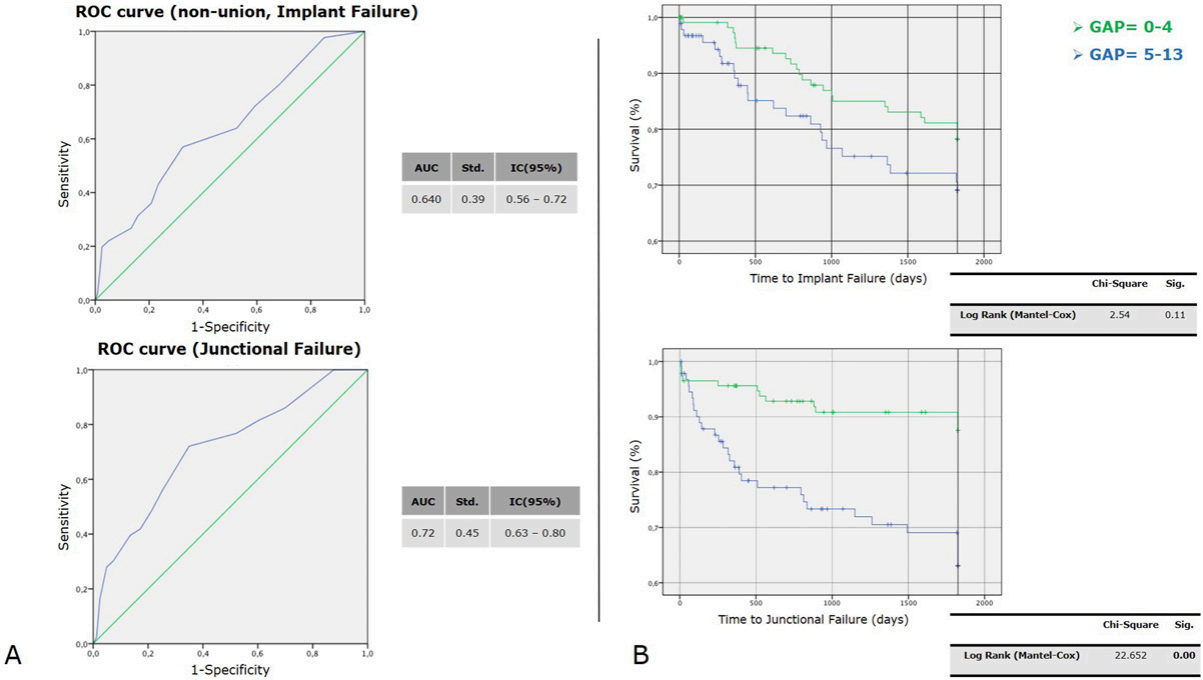
Receiver Operating Characteristic (ROC) curves and Kaplan-Meier survival analyses. (A) ROC curves for predicting non-union/implant failure (top) and junctional failure (bottom), with area under the curve (AUC) values and 95% confidence intervals indicated. (B) Kaplan-Meier survival curves comparing GAP scores (0–4 vs. 5–13) for time to implant failure (top) and junctional failure (bottom). Statistical significance is assessed using the Log Rank (Mantel-Cox) test.

Through the ROC curve, we found that higher post-operative GAP scores were associated with an increased risk of revision for junctional failures (AUC = 0.72 [IC 95%] 0.63 – 0.80), but a less strong association was found for implant failures (AUC = 0.64 [IC 95%] 0.56 – 0.72) (Figure 8(A)).

The Kaplan-Meier method provided an analysis of survival curves for the 2 GAP categories, considering revision surgery for implant failure and junctional failure as the dependent outcomes (Figure 8(B)).

### Univariate Analysis - Roussouly Type Restoration

**Figure 9.**
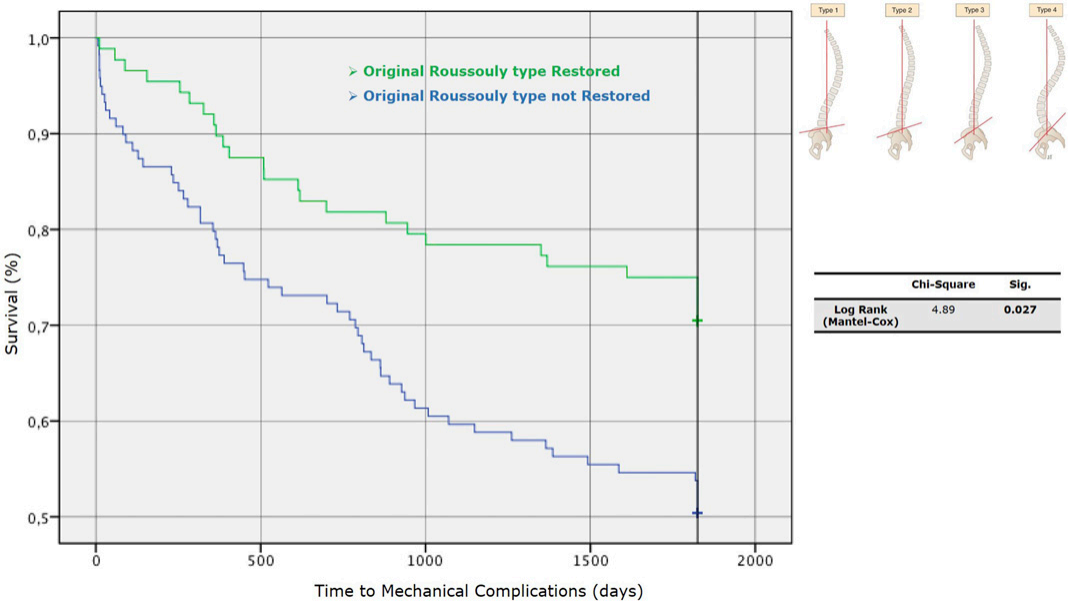
Survival curves for patients with and without restoration of Roussouly type in the absence of mechanical complications (MC).

**Figure 10.**
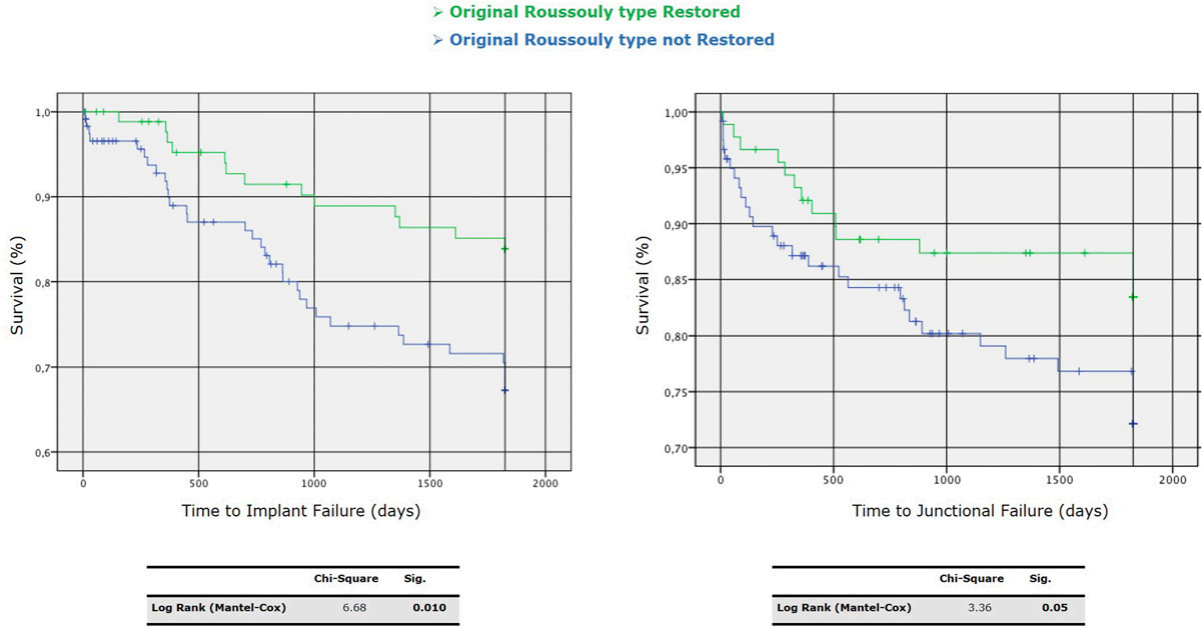
Kaplan-Meier survival curves for time to implant failure (left) and junctional failure (right) comparing patients with restored vs. non-restored original Roussouly type. Statistical significance is indicated by *P*-values of 0.010 for implant failure and 0.05 for junctional failure.

We performed a univariate analysis between Roussouly type restored and not. Through the Kaplan-Meier method, we draw the survival curves for patients with and without restoration of Roussouly type in the absence of MC (Figure 9). Log-rank tests showed statistically significant differences between these curves with a *P* = 0.027 - [χ^2^ (N = 212) = 4.89]. The Kaplan-Meier method provided analysis of survival curves for the Roussouly type restored and not by considering the revision surgery for implant failure and junctional failure as the dependent outcomes (Figure 10).

### Multivariate Analysis - Machine Learning Approach (Step II)

**Figure 11.**
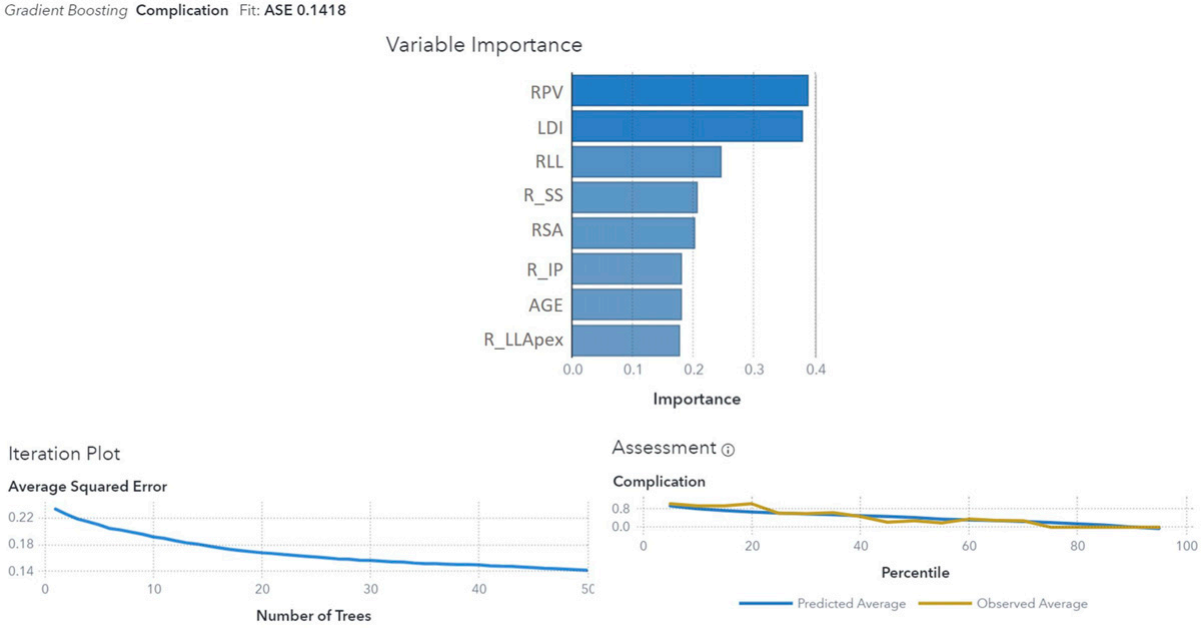
Gradient Boosting model results for predicting complications. The top panel shows the variable importance, with RPV and LDI being the most influential predictors. The iteration plot (bottom left) indicates the model's decreasing average squared error with the increasing number of trees. The assessment plot (bottom right) compares the predicted and observed average complication rates across percentiles, showing good model fit with an ASE of 0.1418.

Among all the ML models, again, the Gradient Boosting showed the higher reliability (ASE 0.14) and allowed us to focus on the correlation between variables and their importance in defining the final output (Figure 11): Relative Pelvic Version, Lordosis Distribution Index and Relative Lumbar Lordosis seem to be the most reliable variables in order to predict MC.

## Discussion

Adult deformity correction requires a challenging and heavy surgery that is related to a high rate of postoperative complications and the need for revision surgery, with a consequent decrease in life quality. Among them, implants and junctional failures are the most common causes of revision surgery, with frequent need for complex procedures,^13^ and strategies to reduce their rate should be implemented. Literature is quite heterogeneous on the possible causes of these types of failures. However, different variables have been found as possible predictors of IF and JF, and many studies proposed algorithms and classifications to reduce the risk of postoperative mechanical complications.

The Roussouly classification, while describing different shapes of normal spines, offers a guide for restoring a pre-degenerative alignment, even with the difficulties linked to identifying the original Roussouly type. On the other hand, once the postoperative alignment is obtained, the GAP score offers a predictive tool to evaluate the risk of mechanical complications in that specific patient, substantially based on global alignment, lordosis distribution and pelvic orientation.

Our study identified a GAP score of 4 as the threshold for increased risk of revision surgery, with higher scores linked to more frequent junctional failures. Patients with significant post-operative deviations in spinopelvic alignment faced greater risks of mechanical complications. Restoration of the original Roussouly type was crucial, as those with restored spinal curvature experienced fewer issues. Personalized surgical planning and precise alignment were emphasized to improve long-term outcomes and reduce post-operative complications.

The aim of our work was not merely to validate a score but to identify key criteria for reducing the risk of reoperations due to mechanical complications. While many risk factors are not modifiable, bone quality remains an important element, as low Hounsfield unit values on preoperative CT scans have been linked to an increased risk of Proximal Junctional Kyphosis (PJK).^14^ Similarly, body mass index (BMI) has been proposed as a relevant predictor of mechanical failure,^15^ leading Noh et al to suggest its inclusion in Yilgor’s predictive score.^16^ Other studies have highlighted additional patient-related factors, such as comorbidities, neurological disorders, and smoking, as contributors to mechanical failure.^17^ Moreover, the quality of paraspinal muscles may influence spinal sagittal balance and postoperative outcomes.^18^ A comprehensive preoperative assessment, particularly of modifiable factors like bone quality and BMI, is essential to optimize surgical outcomes, even though certain frailty-related risks may be difficult to mitigate despite preoperative interventions.^19^

Once modifiable risk factors have been treated, and the patient has been considered eligible for surgery, proper surgical treatment should aim to restore an adequate sagittal and coronal balance,^20^ which has been shown to lead to a reduction in the complication rate. Many authors proposed that if the concept of conus of economy is respected with the head gravity line falling over the feet, the risk of mechanical complications is reduced despite the eventual compensatory mechanism adopted by the patient.^21^ Some studies suggest that elderly patients may not tolerate high-grade corrections and that keeping a ‘compensated imbalance’ might be sufficient to obtain a good Health-Related Quality of Life (HRQoL).^22^ On the other hand, the restoration of an ideal shape of the spine based on Pelvic Incidence value has been found to be a protective factor against complications in different studies: since it was first published in 2017, the GAP score has been considered a significant help in predicting and reducing mechanical complication rates. Several studies recognize a predictive value on mechanical complications with a mean AUC value of 0.79; Jacobs et al^23^ compared the predictive capabilities of the GAP score and Schwab’s sagittal modifiers for mechanical complications, assessing a greater predictive power of the first, even if the study results were limited by the small sample size (39 patients). Dae-Woong Ham et al showed promising results regarding the GAP score’s predictive power for MC and PJF in an elderly population of 84 patients with degenerative kyphoscoliosis.^24^ A recent systematic review of the literature states that, with a mean AUC of 0.68, the GAP score is for the time being only moderately accurate in predicting MC,^25^ and the same findings have been found by many other authors.^26-28^ An important aspect is that the authors globally consider the risk of MC and only in few cases stratify it by type of complication; Sun et al found higher correlations between GAP score values and PJK/PJF occurrence (AUC respectively 0.86 and 0.72) than between GAP score and MC occurrence in general. Our findings seem to confirm the association with the occurrence of JF with an AUC of 0.7, with less influence on whole MC and IF; besides, analyzing the Kaplan-Meyer curves, in the group of patients who showed a postoperative GAP score ≤4, the survival rate in absence of JF at 5-year-FU was around 90%.

Roussouly classification and GAP score are, however, two distinct entities, as the first doesn’t consider the implications of deformity as it’s purely qualitative and descriptive of a physiological sagittal alignment, while the GAP score doesn’t consider the original shape of the spine, which may lead to mistakes in directing the correction where is needed and in properly calibrating the sagittal correction. Each spine had its own anatomy before the onset of the deformity, which is not standardizable and has to be considered and, as much as possible, respected when planning a correction surgery. Moreover, the prediction accuracy of the GAP score for IF could be low, given that material properties (elastic modulus) and the overall structure of the internal fixation system itself are not considered. Finally, the dynamic and living habits of patients can impact hardware-related complications or junctional failures.^29^

In recent publications, several authors have started to stress the importance of considering the original shape of the spine according to the Roussouly Classification^8,30^ and how this restoration has a protective effect on implants in terms of reduction of mechanical complications. As previously underlined the Roussouly classification describes the physiological alignment of spine in asymptomatic subjects, without considering aging, deformity and their implications in terms of compensating mechanisms.^31^ On the other hand, the effects of deformity on spinal alignment and pelvic orientation make it complex to define the original Roussouly type in adult deformity patients. Being the PI value considered constant in adult subjects and being this strictly related to SS values (that are at the foundation of Roussouly’s classification), we can suppose a correspondence between PI and the original spinal degenerative shape; for this reason a PI-based algorithm could be the solution to identify preoperatively the original Roussouly type.^32^ Many studies analyzed the effect of surgical restoration of a proper Roussouly spinal shape on postoperative mechanical complication rate, and in all of them, a positive correlation was found. Bari et al^33^ concluded that surgical correction of ASD in accordance to the ideal Roussouly spine shape is correlated to a substantial significant decrease in risk of revision surgery due to mechanical failure in a cohort of 233 patients; in the paper, however, the rules used to consider the spinal shape as ‘restored’ were not so well described. Conversely, Sebaaly et al^34^ analyzed 290 patients, and they assessed that restoring the sagittal spinal contour to the normal shapes of Roussouly (according to PI) could serve as a guideline for ASD treatment. The suggested algorithm proposed to restore the low-PI patients (PI < 50°) indifferently to type 1 or 2, while higher-PI patients (PI > 50°) are indicated to a restoration of type 3 or 4, indifferently.

In our opinion, types 1 and 2 are too different entities to be grouped together in the analysis. Moreover, different types correspond to different lordosis distributions, and it has been recently published that a proper restoration of lordosis apex seems to reduce the risk of PJK by a ratio of 4.6.^35^

We think the algorithm we propose to evaluate the original Roussouly type in adult patients is clear and reproducible, with a good intra- and interobserver agreement, and gives emphasis to both the pelvis and the spinal shape, taking into account all the aspects of the original classification (Figure 1). A correlation between the restoration of the original Roussouly type and mechanical complication was found, even if GAP score had shown higher predictive value (Table 2).

Analyzing independently all the subgroups of the GAP score and the different types of Roussouly classification, the parameters that mainly influence the MC rate are the relative pelvic version (RPV), the relative lumbar lordosis and lordosis distribution index (LDI) and the relative lumbar lordosis (RLL); planning the surgery, the magnitude of lumbar lordosis and the distribution of lumbar lordosis represent actionable factors that can be managed directly on the surgical field. The pelvic version is not predictable preoperatively because they are influenced by the orthostatic position, but the literature suggests that surgical correction of LL is linearly correlated with the decrease of pelvic retroversion so that the postoperative PT could be hypnotized, restoring the proper lumbar lordosis.^36^

**Figure 12.**
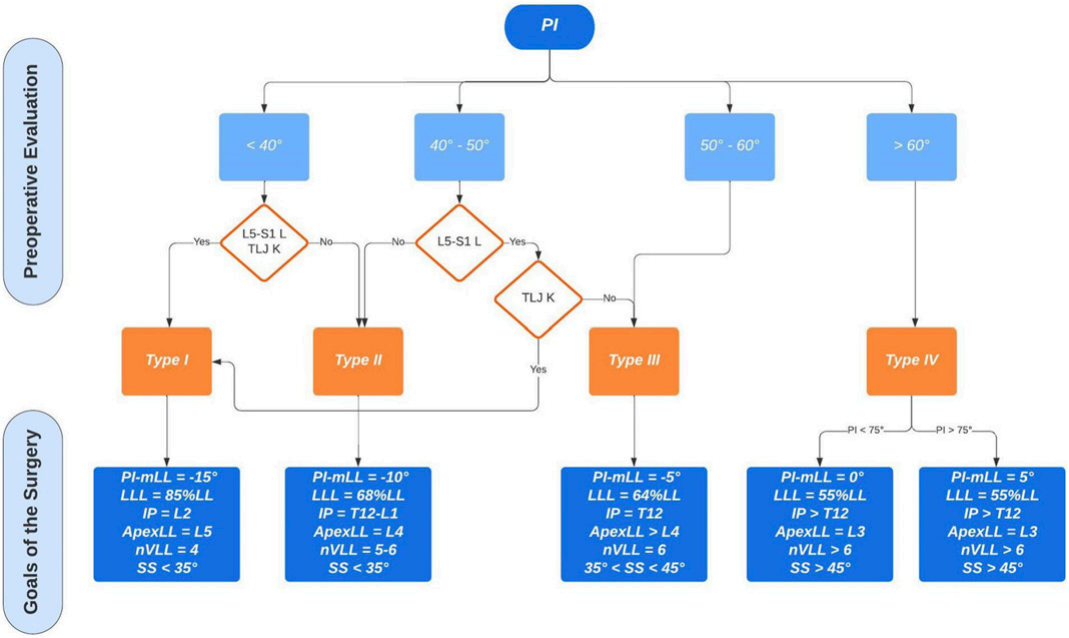
Algorithm to minimize the risk of mechanical failure.

**Figure 13.**
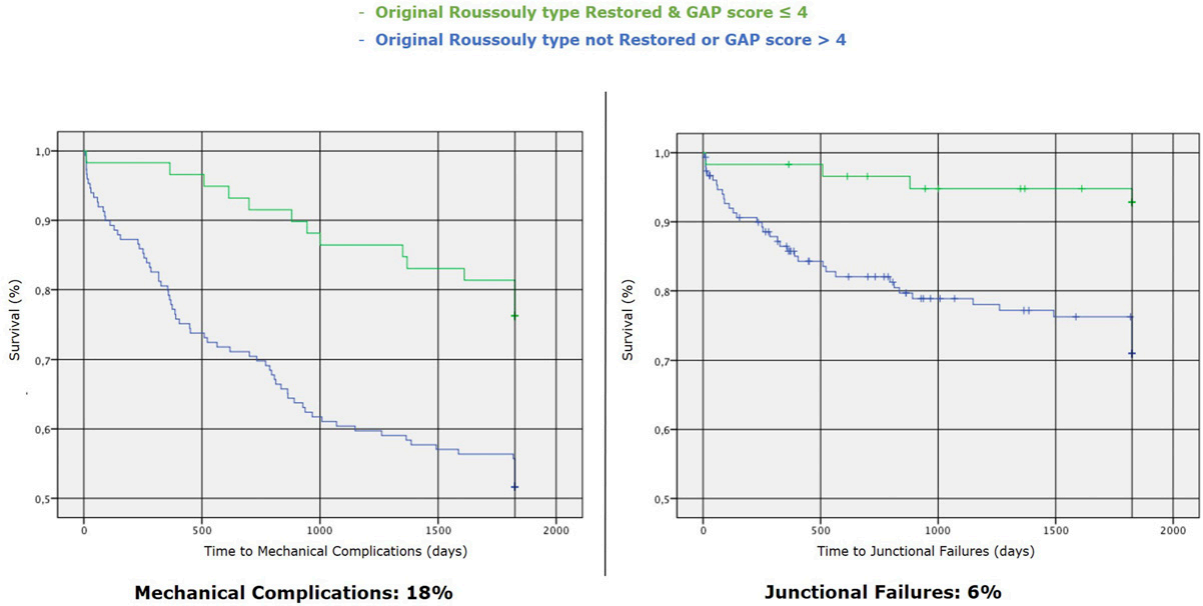
Kaplan-Meier survival curves for time to mechanical complications (left) and junctional failures (right) comparing patients with restored (& GAP score ≤ 4) vs. non-restored (& GAP score > 4) original Roussouly type.

According to the present study’s findings and the factors found in the literature, we propose the algorithm in Figure 12 to minimize the risk of mechanical failure. It considers the PI and the spinal morphology to identify the original shape of the spine; the combination of a qualitative variable (analyzing the spinal shape through Roussouly classification) and a quantitative variable (defining the correction targets to be achieved according to PI through the GAP score) is probably the best way to minimize the risk of mechanical failure and especially junctional failures (depending less on hardware intrinsic factors). In the group of patients who showed a postoperative GAP score ≤4 with a restoration of the original Roussouly type, the MC and JF rate at 5-year-FU were respectively around 18% and 6% (Figure 13).

### Limitation and Strength of the Study

This study has several limitations. First, it’s a retrospective analysis of patients treated for ASD with long FU, so only 212 of the 460 patients could be included in the study because of the strict inclusion and exclusion criteria. Although our analyses showed the baseline characteristics did not differ between patients included in the current study and those not, we cannot exclude a selection bias. Besides, the sample size of GAP score groups is asymmetrical. Third, the HRQoL was not assessed and considered in the analysis, but it does not represent the present study’s focus.

We can consider the strength of our study the long follow-up; it’s a single-centre study, and the analysis includes the spectrum of alignment, according to the fact that the surgeries were performed in different periods (from 2008 to 2016) with different technical knowledge and surgical skills. Besides, all the patients were not previously treated for spinal problems, and all the revision surgeries were not included in the study, and this could have represented a bias, present in most other studies on this topic.

## Conclusion

Since the risk factors for mechanical failure are not all modifiable or predictable, any predictable factor must be evaluated to reduce the complication rate.

The treatment should include adequate patient selection, all preoperative treatments aimed at optimizing comorbidities, and, finally, proper surgery planning and performance.

According to our findings, when planning the surgery, the surgeon should:

- Restore the proper maximum lumbar lordosis: the postoperative lumbar lordosis could be predictable and verifiable during the surgical procedure.
- Consider the proper distribution of lumbar lordosis: according of GAP score, the target of LLL should be 50%-80% of the whole LL. The distribution should consider the original Roussouly type, as recently said by Shen et al^37^:
  • 84.2% (SD 15.7) for Original Type 1
  • 68.3% (SD 14.4) for Original Type 2
  • 64.6% (SD 10.7) for Original Type 3
  • 57.6% (SD 10.8) for Original Type 4
- Avoid pelvic retroversion and achieve a proper global balance: according to the LL planned preoperatively, GT and PT could be hypothesized.

## Data Availability

The datasets used and/or analyzed in the present study are available from the corresponding author upon reasonable request.*
